# Radium‐223 dichloride treatment in metastatic castration‐resistant prostate cancer in Finland: A real‐world evidence multicenter study

**DOI:** 10.1002/cam4.5262

**Published:** 2022-09-26

**Authors:** Anniina Hyväkkä, Okko‐Sakari Kääriäinen, Tapio Utriainen, Eliisa Löyttyniemi, Kalle Mattila, Petri Reinikainen, Jorma Sormunen, Minna Jääskeläinen, Päivi Auvinen, Heikki Minn, Maria Sundvall

**Affiliations:** ^1^ Cancer Research Unit, Institute of Biomedicine, and Department of Oncology FICAN West Cancer Center, University of Turku, Turku University Hospital Turku Finland; ^2^ Department of Oncology Kuopio University Hospital Kuopio Finland; ^3^ Helsinki University Hospital Comprehensive Cancer Center Helsinki Finland; ^4^ Department of Biostatistics, Institute of Clinical Medicine University of Turku Turku Finland; ^5^ Department of Oncology and FICAN West Cancer Center University of Turku, Turku University Hospital Turku Finland; ^6^ Department of Oncology Tampere University Hospital Tampere Finland; ^7^ Docrates Cancer Center Helsinki Finland; ^8^ Department of Oncology Oulu University Hospital Oulu Finland; ^9^ Department of Oncology Lapland Central Hospital Rovaniemi Finland

**Keywords:** bone metastases, castration‐resistant prostate cancer, radium‐233 (Ra‐223) therapy, real‐world evidence

## Abstract

**Background:**

Radium‐233 dichloride is an alpha emitter that specifically targets bone metastases in prostate cancer. Results of a previously reported phase III randomized trial showed survival benefit for radium‐223 compared to best supportive care in castration‐resistant prostate cancer (CRPC) with bone metastases. However, real‐world data are also needed with wider inclusion criteria.

**Methods:**

We report results of a retrospective multicenter study including all patients with metastatic CRPC treated with radium‐223 in all five university hospitals in Finland since the introduction of the treatment. We identified 160 patients who had received radium‐223 in Finland in 2014–2019.

**Results:**

The median overall survival (OS) was 13.8 months (range 0.5–57 months), and the median real‐world progression‐free survival (rwPFS) was 4.9 months (range 0.5–29.8 months). Alkaline phosphatase (ALP) values within the normal range before and during the radium‐223 treatment or the reduction of elevated ALP to normal range during treatment were associated with better OS when compared to elevated ALP values before and during treatment (*p* < 0.0001). High prostate‐specific antigen (PSA) level (≥100 μg/L) before radium‐223 treatment was associated with poor OS compared to low PSA level (<20 μg/L) (*p* = 0.0001). Most patients (57%) experienced pain relief. Pain relief indicated better OS (*p* = 0.002). Radium‐223 treatment was well tolerated. Toxicity was mostly low grade. Only 12.5% of the patients had grade III–IV adverse events, most commonly anemia, neutropenia, leucopenia, and thrombocytopenia.

**Conclusion:**

Radium‐223 was well tolerated in routine clinical practice, and most patients achieved pain relief. Pain relief, ALP normalization, lower baseline PSA, and PSA decrease during radium‐223 treatment were prognostic for better survival. The efficacy of radium‐223 in mCRPC as estimated using OS was comparable to earlier randomized trial in this retrospective real‐world study. Our results support using radium‐223 for mCRPC patients with symptomatic bone metastases even in the era of new‐generation androgen receptor‐targeted agents.

## Graphical abstract

We report results of a retrospective real‐world multicenter study including all patients with metastatic castration resistant prostate cancer (mCRPC) treated with radium‐223 in all five university hospitals in Finland since the introduction of the treatment. In our cohort, PSA decrease, ALP normalization and pain relief correlated with improved survival. Our results support using radium‐223 for mCRPC patients with symptomatic bone metastases even in the era of new generation androgen signaling targeting agents.

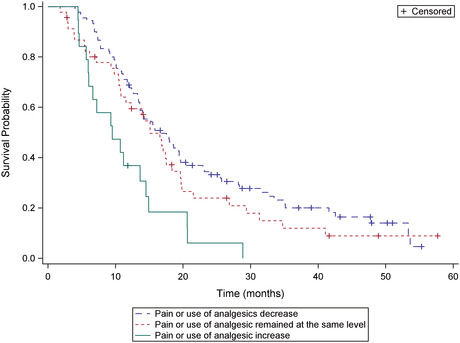

## 1 INTRODUCTION

Prostate cancer is the second most commonly diagnosed cancer in men and sixth leading cause of cancer‐related death among men globally, but even more common cause of cancer‐related mortality in Western countries.^1^ The 5‐year survival rate for local or regional prostate cancer is over 90%, but metastatic disease remains incurable. Bone and lymph node metastasis are typical in prostate cancer, but occasionally also visceral or other metastases may be present.^2^ Androgen‐deprivation therapy (ADT) is the cornerstone for the treatment of metastatic prostate cancer due to the high dependence of prostate cancer cells on androgen signaling. Docetaxel and drugs interfering with androgen signaling by directly binding to androgen receptor (AR) or inhibiting CYP17A involved in androgen synthesis have increased survival in combination with ADT.^3^, ^4^, ^5^, ^6^ However, the majority of the patients eventually develop castration‐resistant prostate cancer (CRPC). Treatments showing benefit in mCRPC include taxanes, androgen signaling inhibitors, and radioisotopes, such as radium‐223 dichloride and lutetium‐177‐PSMA‐617.^4^, ^5^, ^6^, ^7^ Patients with DNA damage repair (DDR) pathway mutations may also benefit from PARP inhibitors.^8^ Supportive therapies include bisphosphonates, denosumab, and palliative external beam radiation therapy.^4^, ^5^, ^6^

Radium‐223 is an α‐emitting radionuclide with a half‐life of 11.4 days, and it is administered as an intravenous injection a maximum of six times every 4 weeks to treat mCRPC predominantly limited to bone.^9^ Bone metastases are often symptomatic and cause pain, bone marrow suppression, hypercalcemia, and skeletal‐related events, such as spinal cord compression or pathological fractures. Therefore, efficient treatment of bone metastases is essential to preserve the quality of life and performance status in mCRPC patients. Radium‐223 dichloride mimics calcium and forms complexes with the bone mineral hydroxyapatite at the areas of high bone turnover such as bone metastases.^10^ The high energy α‐particles emitted by radium‐223 (tissue range < 100 μm) induce breaks to DNA, cell cycle arrest, and eventually death of cancer cells and suppression of tumor‐induced pathologic bone formation.^11^, ^12^, ^13^ Radium‐223 treatment was shown to be safe and efficacious in the phase III ALSYMPCA trial.^14^ The study consisted of 921 patients with mCRPC with predominantly bone metastatic disease. The OS was significantly longer with radium‐223 compared to placebo (median 14.9 vs. 11.3 months, HR, 0.70; *p* < 0.001). All the main secondary end points also showed a benefit of radium‐223 when compared with placebo. Time to first symptomatic skeletal event (median 15.6 vs. 9.8 months, HR, 0.66; *p* < 0.001), time to increase in total ALP (median 7.4 vs. 3.8 months, HR, 0.17; *p* < 0.001), as well as time to increase in PSA level (median 3.6 vs. 3.4 months, HR, 0.64; *p* < 0.001) were significantly longer with radium‐223 as compared to placebo. Importantly, the quality of life was also better in the radium‐223 group.^15^

Since ALSYMPCA trial, several new treatments, including new generation hormonal agents and PARP inhibitors have entered treatment recommendations, and radium‐223 is currently investigated in these settings and in combination with other drugs. The combination of radium‐223 and abiraterone did not improve the symptomatic skeletal event‐free survival and was associated with increased frequently of bone fractures compared to placebo in mCRPC in the ERA‐223 trial.^16^ Ongoing phase III studies evaluate radium‐223 in mCRPC in combination with either with enzalutamide or darolutamide (NCT02194842; NCT04237584) as well as with docetaxel (NCT03574571). In addition, radium‐223 is evaluated in mCRPC in phase I and II trials in combination with different immune checkpoint inhibitors (pembrolizumab, NCT03093428; nivolumab, NCT04109729; avelumab, NCT04071236 and PARP inhibitors [niraparib NCT03076203; olaparib NCT03317392]).

More real‐world evidence (RWE) regarding the safety and efficacy of radium‐223 in routine clinical practice is needed, because the ALSYMPCA trial was finished before the newer generation hormonal treatments became widely used in mCRPC. The aim of this retrospective study was to evaluate the efficacy and safety of radium‐223 treatment and to identify which patients have benefitted most from radium‐223 treatment in a real‐world setting in Finland since the introduction of this therapy.

## 2 MATERIALS AND METHODS

### 2.1 Patient population

We retrospectively collected data of all patients with metastatic castration resistant prostate cancer (mCRPC) and bone metastases treated with injections of radium‐223 dichloride in all five university hospitals in Finland (Helsinki, Tampere, Turku, Kuopio, Oulu) since 2014 until the end of 2019. Patients treated in clinical trials were excluded.

### 2.2 Study design

The primary endpoints of this retrospective analysis were to evaluate (1) OS and (2) skeletal pain response (changes of use of analgesics) of patients with mCRPC treated with radium‐223 in Finland.

The main secondary endpoints were (1) to analyze the changes of PSA and ALP levels during radium‐223 treatment; (2) to analyze progression of disease during radium‐223 treatment by radiological imaging; (3) to evaluate the real‐world progression‐free survival (rwPFS); (4) to analyze predictive factors of OS; (5) to evaluate safety; and (6) to evaluate causes of early discontinuation of the treatment.

### 2.3 Data collection

Data were collected retrospectively by investigators from the original medical records of Helsinki, Tampere, Turku, Kuopio, and Oulu University Hospitals. Demographic data included the date of the initial diagnosis for prostate cancer, Gleason score, primary PSA value, the date of metastatic disease, the date of CRPC, and the date of treatment decision to start radium‐223. Charlson Comorbidity Index, metastatic sites, ECOG performance status, and the use of analgesic drugs were collected at the time of the initiation of radium‐223. Treatments for prostate cancer before and after radium‐223 (radiotherapy, surgery, docetaxel, cabazitaxel, abiraterone, enzalutamide, palliative radiotherapy), and the use of bisphosphonates or denosumab were obtained. We collected the information of radium‐223 therapy including dates, the number of cycles delivered, and relevant laboratory values (PSA, ALP, hemoglobin, leukocytes, neutrophils, thrombocytes). Adverse events during radium‐223 treatment were graded according to the Common Terminology Criteria for Adverse Events (CTCAE, version 5.0). The information on radiological imaging, the date of disease progression, skeletal‐related events, the reason for early discontinuation of radium‐223 treatment, and survival were collected. rwPFS was defined as the time from the date of decision to start radium‐223 treatment until disease progression or death from any cause. Disease progression was defined based on the recorded assessment of treating physician's judgment on radiology findings and reports, laboratory evidence (PSA), and clinical assessment, or start of a new treatment line after radium‐223. Pain response during radium‐223 treatment was assessed by changes of the use of analgesics (group 1: no analgesics; group 2: NSAID [non‐steroidal anti‐inflammatory drug] or paracetamol; group 3: opioids) or based on recording significant pain relief by treating physician in the original medical records.

### 2.4 Timepoints

All laboratory values in this study have been collected before the first treatment of radium‐223, before each treatment cycle and at the end of the treatment. Radiologic imaging has been evaluated in the middle of the treatment (usually after 3. cycle) and at the end of the treatment. Pain response was evaluated before the first treatment of radium‐223, during of the treatment and at the end of the treatment.

### 2.5 Response assessment

Response of the treatments was assessed according to the change in disease‐related biomarkers PSA and ALP, and radiologically according to an original assessment of radiological images in CT or bone scintigraphy. Pain response was evaluated during and after treatment as defined earlier.

### 2.6 Statistical analysis

OS was calculated from the date of treatment decision to start radium‐223 to the date of death of any cause. Definition of rwPFS and disease progression are described earlier. OS and rwPFS were estimated according to the Kaplan–Meier method. Patients were followed until July 31, 2020. Patients who were still alive at the time of analysis were censored at the last available date on which they were known to be alive before the end of follow‐up time. Categorical variables are summarized with counts and percentages, continuous variables with median and range. Survival analyses were started with Kaplan–Meier curve and log‐rank test continued with univariate Cox's proportional hazard model. Cox's model examined effect of PSA change as well as PSA baseline level, amount of treatment lines, pain response, ALP response, number of radium‐223 cycles, ECOG performance status, time from metastases to the initiation of radium‐223 therapy. Univariate approach was chosen while these factors are naturally correlated. The significance level of 0.05 (two‐tailed) was used for statistical significance. The data analysis for this paper was generated using SAS software, Version 9.4 of the SAS System for Windows (SAS Institute Inc., Cary, NC, USA).

### 2.7 Ethical consideration

The study was approved by the institutional research boards (License numbers TO6‐054‐19‐ T273‐2018, T06‐041‐21‐T263‐2021; HUS/824/2020; R20524; OYS‐8/2020; 5,654,232) and the Social and Health Data Permit Authority, THL/6958/14.02.00/2020 findata‐rems‐2020/753. Informed consent was waived due to retrospective design of the study according to Finnish act on Secondary Use of Social and Health Data effective from April 2019 (Act 552/2019). All data were collected, stored, and handled in a manner that meets the regulation of GDPR and the Secondary Use Act 552/2019. The data that support the findings of this study are available on request from the corresponding author. The data are not publicly available due to privacy restrictions and regulations mandated by the Secondary Use Act.

## 3 RESULTS

### 3.1 Population

Since radium‐223 treatment was introduced in Finland in 2014, 160 patients have received radium‐223 at all 5 university hospitals of Finland by the end of 2019. The median age at the date of treatment decision to start radium‐223 was 72 years (range, 53–95 years) and most of patients were in good general condition (ECOG 0–1 = 119 pts, 74%) before first radium‐223 cycle. The median baseline PSA value before the first treatment of radium‐223 was 92 μg/L (range, 3.4–3363 μg/L) and the median ALP value was 122 U/L (range, 31–1375 U/L; reference value range, 35–105 U/L). The majority of patients (124 pts, 78%) had only skeletal metastases at the time of primary metastasis. The rest of 34 patients (21%) had bone and lymph node metastases. Patient demographics and baseline characteristics are outlined in Table 1.

**TABLE 1:** Baseline characteristics of the patient

Age (years) at initial diagnosis of prostate cancer	
Median (range)	65 (49–93)
>75 year (*n*, %)	15 (9)
Gleason score at initial diagnosis of prostate cancer (*n*, %)	
≤ 6	14 (9)
7	35 (22)
≥ 8	96 (60)
Unknown	15 (9)
PSA (μg/L) level at primary diagnosis	
Median (range)	40 (2.7–5000)
Age (years) at diagnosis of metastatic prostate cancer	
Median (range)	67 (50–94)
Age (years) at diagnosis of CRPC	
Median (range)	70 (52–94)
Age (years) at the date of treatment decision to start radium‐223 treatment	
Median (range)	72 (53–95)
Concomitant diseases, Charlson comorbidity scale (*n*, %)	
6	68 (43)
7	39 (24)
8	28 (18)
9	15 (9)
≥ 10	8 (5)
Unknown	2 (1)
Metastases (*n*, %)	
Bone only	124 (78)
Lymph node + bone	34 (21)
Not evaluable^a^	2 (1)
Initial ECOG performance status prior to first radium‐223 cycle (*n*, %)	
0	17 (11)
1	102 (64)
2	32 (20)
3	5 (3)
Unknown	4 (3)
Use of analgesic before radium‐223 treatment (*n*, %)	
No	26 (16)
Only NSAIDs or paracetamol	34 (21)
Opioids	98 (61)
Unknown	2 (1)

Abbreviations: CRPC, castration‐resistant prostate cancer; ECOG, The Eastern Cooperative Oncology Group, performance status; NSAIDs, non‐steroidal anti‐inflammatory drugs; PSA, prostate‐specific antigen.

^a^ Data to evaluate if metastasis outside of bone were present were not available.

### 3.2 Previous treatments

The majority of patients had received previous therapy with curative‐intent for prostate cancer (radiation therapy: 80 pts, 50% and prostatectomy+/−lymphadenectomy: 34 pts, 21%). All patients had received ADT for metastatic castration‐naïve prostate cancer. Most of the patients had received other treatments for metastatic castration‐resistant prostate cancer (mCRPC) before radium‐223, including docetaxel (115 pts, 72%), cabazitaxel (29 pts, 18%), or androgen signaling inhibitors (abiraterone 98 pts, 61%; enzalutamide 91 pts, 57%). Only 25 (16%) patients received radium‐223 treatment in first line for mCRPC. The majority of patients had received two or three lines of therapy before radium‐223 (one line 19 pts, 12%; two or three lines 87 pts, 54%; more than three lines 29 pts, 18%). Sixty‐three percent of patients (100 pts) had got external beam radiotherapy for painful metastasis and almost all of patients (153 pts, 96%) received bisphosphonates or/and denosumab before radium‐223. Data from previous treatments are presented in Table 2.

**TABLE 2:** Treatments and laboratory values before radium‐223 treatment

Treatments before radium‐223 (*n*, %)	
Prostatectomy	34 (21)
Radical radiotherapy	80 (50)
Docetaxel	115 (72)
Cabazitaxel	29 (18)
Abiraterone	98 (61)
Enzalutamide	91 (57)
Other^a^	29 (18)
Palliative radiotherapy for bone pain before radium‐223 treatment (*n*, %)	
Yes	100 (63)
No	60 (38)
Use of bisphosphonates or/and denosumab before radium‐223 treatment (*n*, %)	
Yes	153 (96)
No	4 (3)
Unknown	3 (2)
Radium‐223 as first line of therapy for CRPC (*n*, %)	25 (16)
One line of therapy before radium‐223	19 (12)
Two lines of therapy before radium‐223	45 (28)
Three lines of therapy before radium‐223	42 (26)
Four lines of therapy before radium‐223	20 (13)
Five lines of therapy before radium‐223	9 (6)
Laboratory tests prior to first radium‐223 cycle (median, range)	
ALP (U/L) (reference range 35–105) (*n*, %)	
< 35	2 (1)
35–105	62 (39)
> 105	88 (55)
Unknown	8 (5)
Median (range)	122 (31–1375)
PSA (μg/L)	92 (3.4–3363)
Hemoglobin (g/L) (reference range 134–167)	123 (81–160)
Leukocytes (E9/L) (reference range 3.4–8.2)	6.2 (2.3–13.2)
Neutrophils (E9/L) (reference range 1.5–6.7)	3.6 (1.2–8.8)
Thrombocytes (E9/L) (reference range 150–360)	238 (117–715)

^a^ ^153^Samarium‐EDTMP, combination of mitoxantrone and prednisone, estramustine, docetaxel re‐challenge or in clinical trial darolutamide or combination of abiraterone and enzalutamide.

Abbreviations: ALP, alkaline phosphatase; CRPC, castration‐resistant prostate cancer; PSA, prostate‐specific antigen.

### 3.3 Radium‐223 treatment and responses

The median number of radium‐223 cycles was 4. Only 37% of patients (59 pts) received all six radium‐223 cycles and the majority of patients discontinued treatment earlier (mainly because of disease progression in 56 pts, 35%). Eight percent of patients (12 pts) received only one cycle of radium‐223. The information of radium‐223 treatment and reasons for discontinued radium‐223 treatments are presented in Table 3.

**TABLE 3:** Information about and after radium‐223 treatment

Total number of radium‐223 cycles (*n*, %)	
1	12 (8)
2	18 (11)
3	44 (28)
4	15 (9)
5	12 (8)
6	59 (37)
Median (range)	4 (1–6)
Reason of discontinuation (*n*, %)	
6 cycles of radium‐223	59 (37)
Progressive disease (PD)	56 (35)
Toxicities	34 (21)
Preplanned cycles less than 6 given	4 (3)
Other^a^	5 (3)
Unknown	2 (1)
Treatments after radium‐223 (*n*, %)	
Docetaxel	11 (7)
Cabazitaxel	35 (22)
Abiraterone	25 (16)
Enzalutamide	38 (24)
Other^b^	6 (4)
Palliative radiotherapy for bone pain after radium‐223 treatment (*n*, %)	
Yes	76 (48)
No	84 (53)
Skeletal‐related events (*n*, %)	
Yes	13 (8)
No	139 (87)
Unknown	8 (5)

^a^ Traumatic cerebral hemorrhage (2 patients, no thrombocytopenia), headache/ocular symptoms (2 patients, later orbital or visceral metastases detected), and acute death due to unknown cause after the first radium‐223 cycle.

^b^ ^177^Lutetium‐PSMA, ^153^Samarium‐EDTMP, combination of mitoxantrone and prednisone or estramustine.

Changes of total ALP and PSA levels were analyzed during radium‐223 treatment. ALP level was over upper limit normal (ULN, 105 U/L) in 55% of the patients (88 pts) before radium‐223 treatment but normalized below ULN during treatment in 30% of these patients (26 pts). Overall, ALP levels decreased in almost half (49%) of the patients (78 pts) over 25% during radium‐223 treatment. Only 7% of patients (11 pts) had ALP increased over ULN during treatment (increased was defined as over 25% increase from baseline ALP). PSA level decreased in 20% of the patients (32 pts) during treatment compared to baseline PSA value and 50% of these patients (16 pts) got PSA response (PSA decrease ≥50%). PSA was stable (increase <25%) in 8% of patients (12 pts) during treatment. However, PSA increased (over 25%) during treatment in 70% of patients (112 pts), and PSA level more than doubled in 59% of these patients (66 pts). Responses to radium‐223 treatments are presented in Table 4.

**TABLE 4:** Responses of radium‐223 treatment

Results of radiological imaging (*n*, %)^a^	
After 3 cycles of radium‐223	
No progressive disease	53 (33)
Partial response	3 (2)
Stable disease	50 (31)
Progressive disease	56 (35)
Not evaluable for response	15 (9)
Not determined	36 (23)
After 6 cycles of radium‐223	
No progressive disease	30 (19)
Partial response	1 (1)
Stable disease	29 (18)
Progressive disease	33 (21)
Not evaluable for response	13 (8)
Not determined	84 (53)
Pain response (*n*, %)	
Less pain and/or use of analgesics	91 (57)
Same pain and/or use of analgesics	46 (29)
More pain and/or use of analgesics	19 (12)
Unknown	4 (3)
PSA response (*n*, %)	
Decrease	32 (20)
Response (decrease >50%)	16 (10)
Stabile (increase <25%)	12 (8)
Increase >25%	112 (70)
Increase >100%	66 (41)
Unknown	4 (3)
ALP response (no./total no., %)	
Patients with ≥ 25% reduction in ALP^b^	78/160 (49)
Patients with normalization of ALP^c^	26/88 (30; 16 of total)

Abbreviations: ALP, alkaline phosphatase; PSA, prostate‐specific antigen.

^a^ According to an original real‐world assessment of radiologic images in CT or bone scintigraphy.

^b^ All patient who had ALP reduction ≥25% regardless of primary ALP.

^c^ Only patients who had elevated total alkaline phosphatase levels at baseline are included.

Radiological imaging was generally performed after the third and the last (over 3 cycles) cycle of radium‐223. After three cycles of radium‐223, 35% of patients (56 pts) had progressive disease and 33% of patients (53 pts) had no disease progression radiologically (Table 4). For the rest of the patients, the response was not evaluable or not determined radiologically. After the last cycle (over 3 cycle) of radium‐223, 21% of patients (33 pts) had progressive disease and 19% of patients (30 pts) had no disease progression radiologically. For the rest of the patients, the response was not evaluable or not determined radiologically.

Most of patients (57%, 91 pts) got pain relief with radium‐223 treatment or needed less analgesics than before radium‐223 (Table 4). In 29% of the patients (46 pts), pain or use of analgesic remained at the same level than before radium‐223. Pain or use of analgesic increased in 12% of the patients (19 pts) during radium‐223.

### 3.4 Survival

**FIGURE 1:**
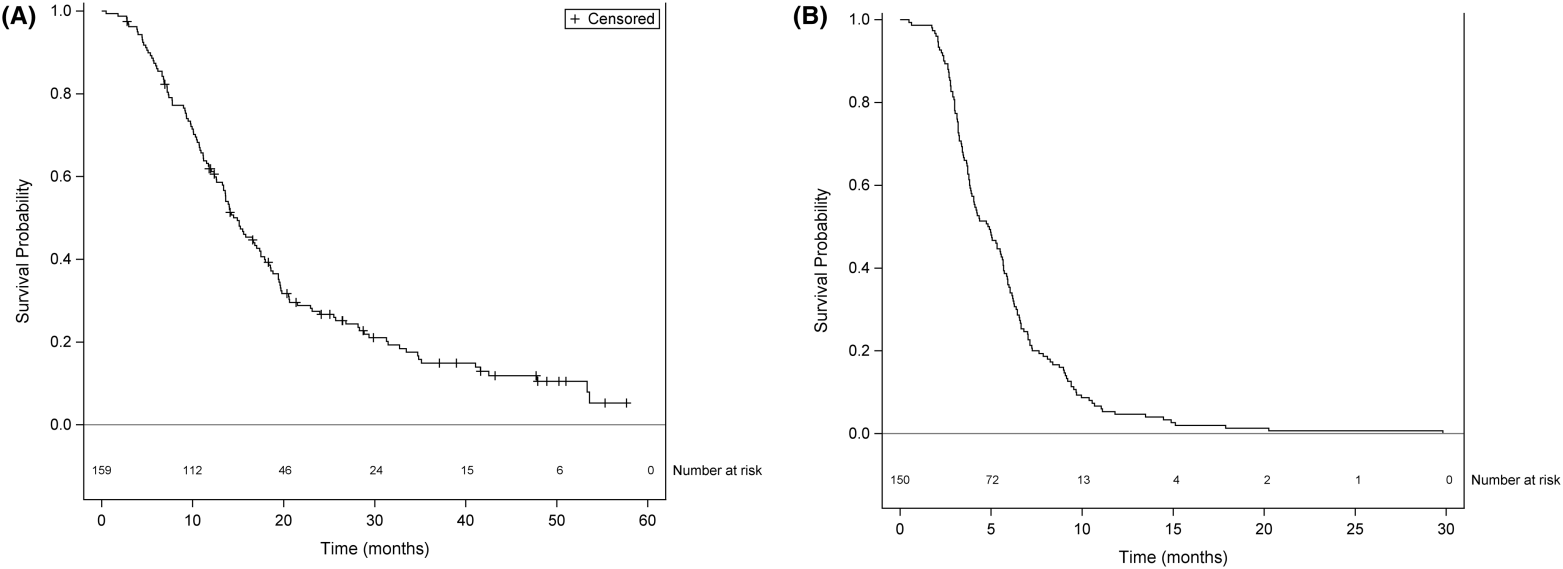
(A) Overall survival (OS) and (B) real‐world progression free survival (rwPFS) of patients treated with radium‐223.

**FIGURE 2:**
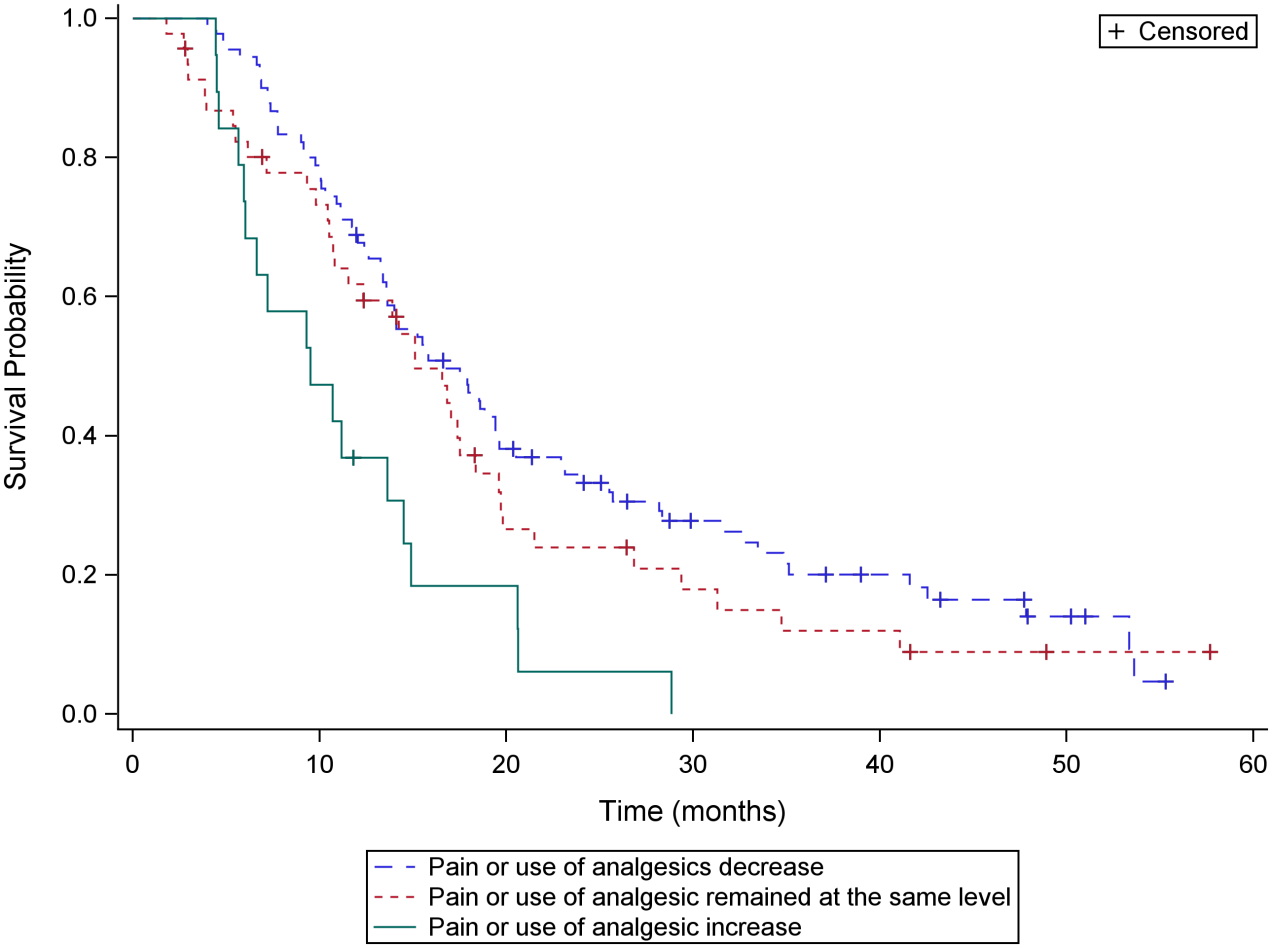
Overall survival (OS) according to the pain response. ‐‐ 1. Pain or use of analgesics decrease. ‐‐ 2. Pain or use of analgesic remained at the same level than before radium‐223. – 3. Pain or use of analgesic increase.

**FIGURE 3:**
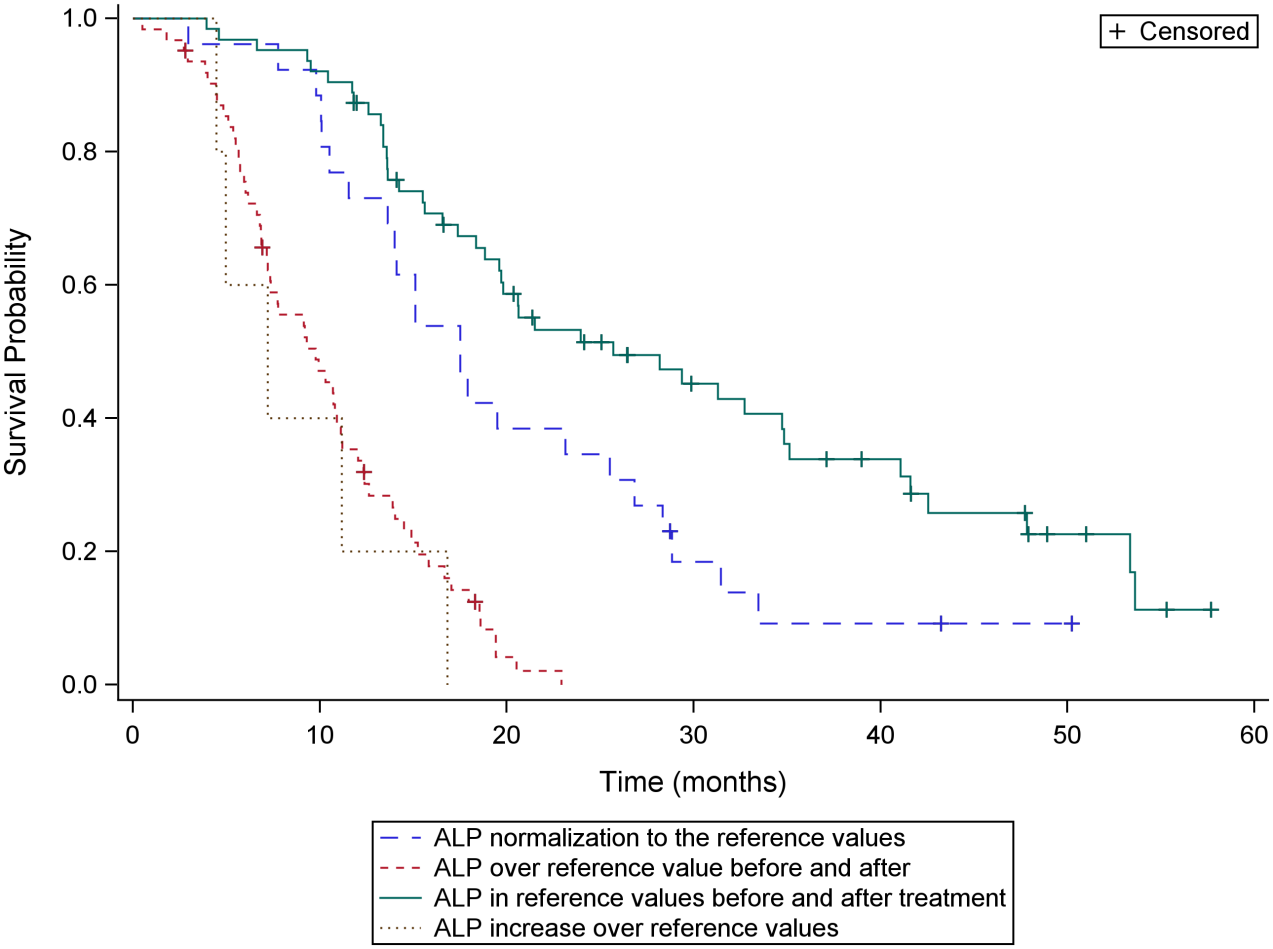
Overall survival (OS) according to the alkaline phosphatase (ALP) response. ‐‐ 1. ALP normalization to the reference values (35–105 U/L) during radium‐223 treatments. ‐‐ 2. ALP over reference value before and after radium‐223 treatment. – 3. ALP in reference values before and after treatment. ‐‐ 4. ALP increase over reference values during radium‐223 treatment.

**FIGURE 4:**
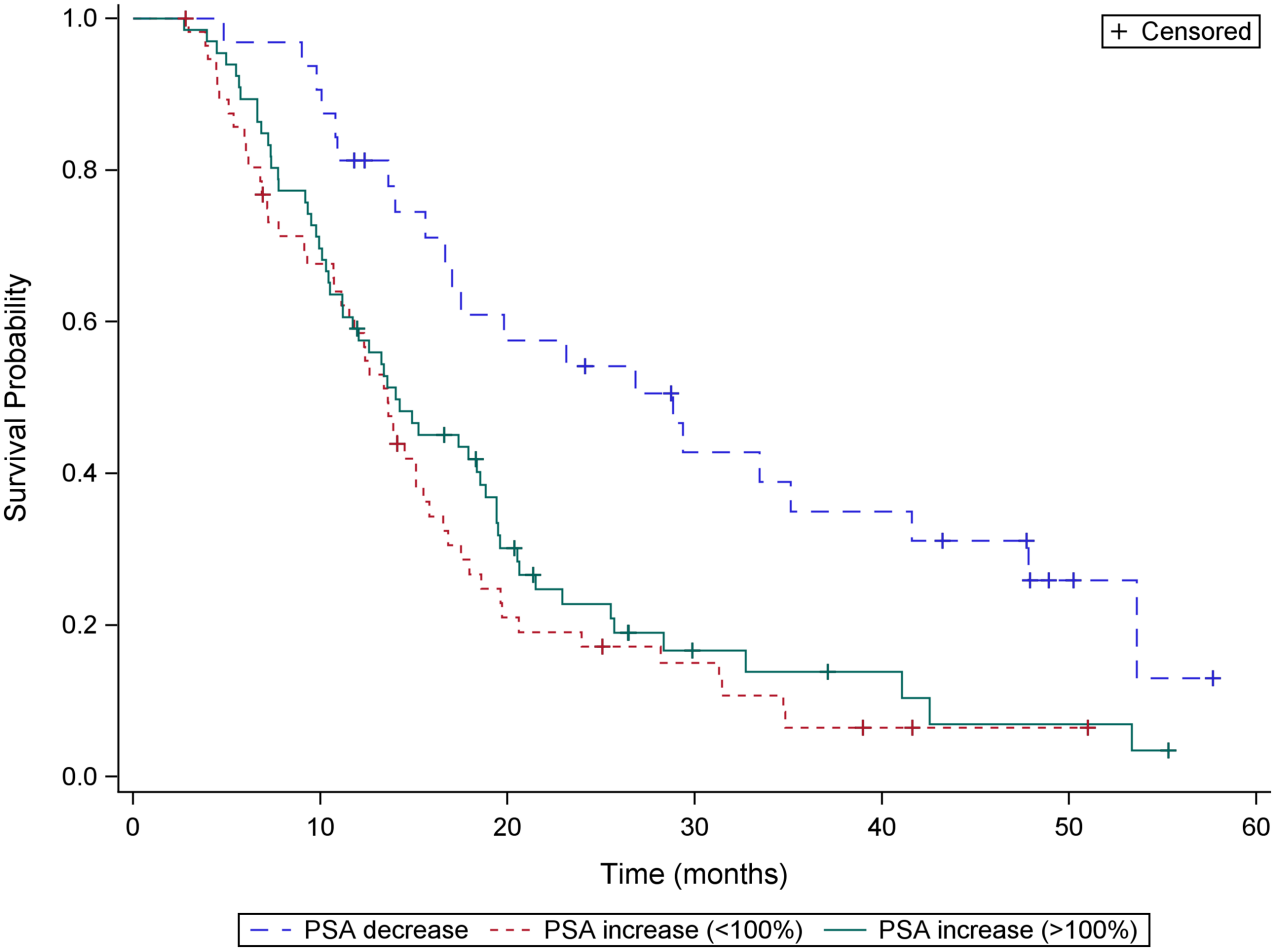
Overall survival (OS) according to the prostate‐specific antigen (PSA) response. – 1. PSA response during radium‐223 treatments. ‐‐ 2. PSA progression less than 100% compared to baseline PSA value during radium‐223 treatment. – 3. PSA more than doubles compared to baseline PSA value during radium‐223 treatment.

**FIGURE 5:**
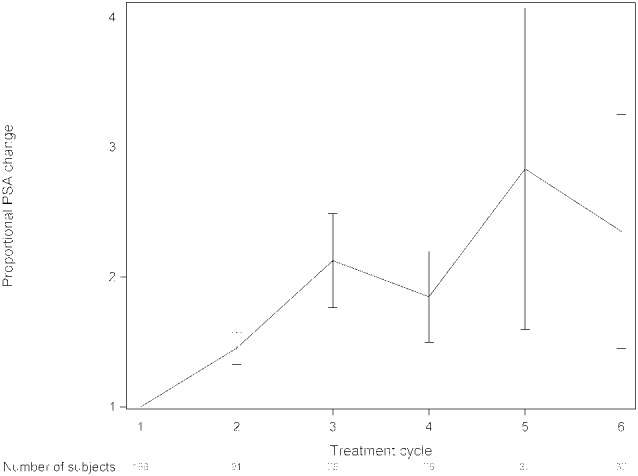
Proportional prostate‐specific antigen (PSA) changes and 95% confidence interval during radium‐223 treatment. PSA was determined before each radium‐223 cycle.

**FIGURE 6:**
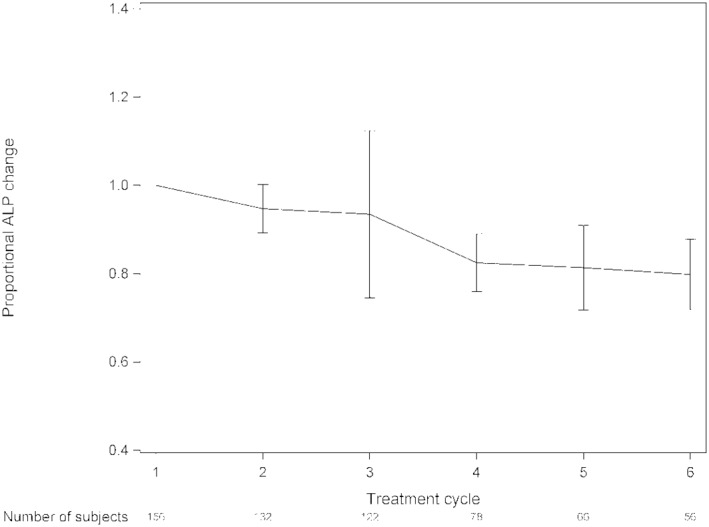
Proportional alkaline phosphatase (ALP) changes and 95% confidence interval during radium‐223 treatment. ALP was determined before each radium‐223 cycle.

The median OS was 13.8 months (range, 0.5–57 months) and median rwPFS was 4.9 months (range, 0.5–29.8 month) (Figure 1). We analyzed survival data according to different parameters. Pain relief was prognostic for survival (overall *p* = 0.002; median OS, 16.0 vs. 14.0 vs. 9.4 months; response vs. same *p* = 0.25; response vs. progression *p* = 0.0005) (Figure 2). The median OS was also longer in patients who achieved ALP response leading to ALP normalization to the reference values with radium‐223 when compared to patients with elevated ALP values before and during treatment (median OS 17.3 vs. 9.1 months, *p* < 0.0001) (Figure 3). The lower the baseline PSA the longer OS was observed (overall *p* < 0.0001; median OS 20.3 vs. 13.2 vs. 8.3 months; PSA ≤20 μg/L vs. PSA 100–500 μg/L, *p* = 0.002; PSA ≤20 μg/L vs. PSA 500–1000 μg/L, *p* < 0.0001). PSA decrease during radium‐223 treatment was also associated with longer OS compared to PSA increase (median OS 23.3 vs. 13.4 months, *p* = 0.0003). However, we did not find a statistically significant difference between PSA progression less than 100% compared to PSA progression more than twofold rise (median OS 13.2 vs. 13.6 months, *p* = 0.34) (Figure 4). Proportional ALP and PSA changes during radium‐223 treatment are presented in Figures 5 and 6.

The number of radium‐223 cycles was prognostic for survival (overall *p* < 0.0001; median OS 5.8 vs. 10.5 vs. 14.3 vs. 23.2 months; 1–2 vs. 3 cycles *p* = 0.002; 1–2 vs. 4–5 cycles *p* < 0.0001; 1–2 vs. 6 cycles *p* < 0.0001). The median OS was shorter in patients with a short time (less than 1 year) from metastases to the initiation of radium‐223 therapy (overall *p* < 0.01; median OS 12.7 vs. 13.4 vs. 19.3 vs. 19.3 months; <1 vs. 2–5 years *p* = 0.03; <1 vs. 5–10 years *p* = 0.004; <1 vs. >10 years *p* = 0.02) indicating possible more aggressive disease. The good ECOG performance status before radium‐223 treatment was prognostic for survival, (overall *p* = 0.01; median OS 17.1 vs. 15.3 vs. 12.7 vs. 9.6 months; 0 vs. 1. *p* = 0.63; 0 vs. 2 *p* = 0.26; 0 vs. 3 *p* = 0.005).

We also analyzed the possible impact of number of treatment lines before radium‐223 on survival but we found no significant difference. Sixteen percent of the patients (25 pts) had received radium‐223 as the first‐line therapy but we found no statistically significant difference in their median OS compared to those who had received radium‐223 as second‐, third‐, fourth‐, fifth‐, or sixth‐line of therapy (overall p = 0.76; median OS 15.0 vs. 14.9 vs. 12.2 vs. 13.1 vs. 14.2 vs. 16.6 vs; 1 vs. 2. *p* = 0.59 1 vs. 3 *p* = 0.57; 1 vs. 4 *p* = 0.36; 1 vs. 5 *p* = 0.96; 1 vs. 6 *p* = 0.88).

### 3.5 Toxicities

Due to the retrospective data collection, toxicity data were not systematically collected. The data presented are based on the data reported in the patient records. The majority of patients had only grade I–II adverse events. Only 12.5% of the patients (20 pts) experienced grade III–IV adverse events, most commonly anemia, neutropenia, leucopenia, and thrombocytopenia. One of the patients experienced a fatal (grade V) thrombocytopenia after radium‐223 therapy. Twenty‐one percent of the patients (34 pts) had to discontinue radium‐223 therapy due toxicities. All adverse events are reported in Table 5.

**TABLE 5:** Adverse events in radium‐223 patients

	*n* = 160					
Adverse Event (*n*, %)	All Grades	Grade I	Grade II	Grade III	Grade IV	Grade V
Hematologic						
Anemia	54 (34)	22 (14)	26 (16)	6 (4)	0	0
Thrombocytopenia	35 (22)	28 (18)	3 (2)	2 (1)	1 (0.6)	1 (0.6)
Neutropenia	35 (22)	0	26 (16)	8 (5)	1 (0.6)	0
Leukopenia	56 (35)	26 (16)	26 (16)	4 (3)	0	0
Nonhematologic						
Diarrhea	13 (8)	11 (7)	2 (1)	0	0	0
Constipation	4 (3)	4 (3)	0	0	0	0
Asthenia (lower limb)	4 (3)	4 (3)	0	0	0	0
Fatigue	16 (10)	14 (9)	1 (0.6)	1 (0.6)	0	0
Bone pain	10 (6)	4 (3)	5 (3)	1 (0.6)	0	0
Nausea	14 (9)	11 (7)	3 (2)	0	0	0
Deterioration in general physical health	6 (4)	6 (4)	0	0	0	0
Infection	4 (3)	2 (1)	2 (1)	0	0	0
Fever	1 (0.6)	1 (0.6)	0	0	0	0
Alanine aminotransferase (ALAT) increase	2 (1)	0	2 (1)	0	0	0
Rash	1 (0.6)	1 (0.6)	0	0	0	0
Medullary compression	1 (0.6)	0	1 (0.6)	0	0	0
Esophagus hemorrhage	1 (0.6)	0	1 (0.6)	0	0	0
Migraine more than before	1 (0.6)	1 (0.6)	0	0	0	0

## 4 DISCUSSION

Radium‐223 is one of the treatment options available for bone predominant mCRPC. Here we report a comprehensive retrospective series of patients treated with radium‐223 in a real‐world setting in Finland during 2014–2019. Results showed that radium‐223 was well tolerated and most patients (57%, 91 pts) achieved pain relief during treatment. The median overall survival (OS) was 13.8 months (range, 0.5–57 months), and the median rwPFS was 4.9 months (range, 0.5–29.8 months). ALP normalization, pain relief, low baseline PSA, and PSA decrease during radium‐223 treatment were prognostic for better survival.

Our data include all 160 patients who have been treated with radium‐223 in all five university hospitals in Finland outside clinical trials. Our unselected retrospective patient cohort is relatively well comparable with the prospective landmark ALSYMPCA trial although our patients had more previous treatment lines before radium‐223 (Table 6). As expected, the median OS in our heavily pretreated real‐world patient cohort was slightly lower than in ALSYMPCA trial (13.8 vs. 14.9 months), but in line with other earlier RWE studies reported from Italy (median OS 14.2 months^17^ or mean OS 10.1 months^18^), the Netherlands (median OS 12.2 months),^19^ and the United Kingdom (median OS 7.3–11.1 months).^20^, ^21^, ^22^, ^23^, ^24^ In our study, patients with good ECOG performance status and who completed all 6 cycles of radium‐223 had significantly longer survival compared to patients with lower ECOG performance status and lower number of treatment cycles. When compared with the ALSYMPCA, the ECOG performance status was slightly lower (ECOG ≥2 23% vs. 13%) in our cohort which is typical in RWE data series, and which could have contributed to the slightly shorter OS observed in our cohort (Table 6). Radium‐223 was also well tolerated in our study.

**TABLE 6:** Patient information compared with the ALSYMPCA randomized trial

	Our retrospective cohort, %	ALSYMPCA trial, radium‐223 arm
Patient, *n*	160	614
Median age before radium‐223 treatment	72 (53–95 years)	71 (49–90)
ECOG 0–1	119 (74%)	536 (87%)
ECOG ≥2	37 (23%)	77 (13%)
Median baseline PSA (μg/L)	92 (range, 3.4–3363)	146 (3.8–6026)
Median baseline ALP (U/L)	122 (range, 31–1375)	211 (32–6431)
Median hemoglobin (g/L)	123 (range, 81–160)	122 (85–157)
Prior docetaxel	115 (72%)	352 (57%)
Use of bisphosphonates or/and denosumab	153 (96%)	250 (41%)
Use of opioids before radium‐223 treatment	98 (61%)	345 (56%)
ALP reduction ≥30%	74 (46%)	233/497 (47%)
Patients with normalization of ALP^a^	26/88 (30%)	109/321 (34%)

^a^ Only patients who had elevated ALP at the baseline are included.

Abbreviations: ALP, alkaline phosphatase; ECOG, The Eastern Cooperative Oncology Group; PSA, prostate‐specific antigen.

We also analyzed disease‐associated biomarkers (PSA and ALP) in our patient cohort during radium‐223 treatment similarly as in the ALSYMPCA trial. We found that normalization of ALP during radium‐223 treatment correlated with longer OS. In our study population, elevated ALP level was observed in 55% of the patients (88 pts) before radium‐223 but ALP levels were reduced to normal range during treatment in 30% of these patients (26 pts). The ALSYMPCA trial showed that significantly higher proportion of patients in the radium‐223 group had an ALP response (reduction ≥30%, *p* < 0.0001) and normalization of ALP level (*p* < 0.0001) than the placebo group.^13^ In our study, the normalization of ALP during radium‐223 treatment was associated with improved OS as observed in the ALSYMPCA trial (Table 6). Also, a retrospective study of 180 mCRPC patients treated with radium‐223 showed similar results, and patients with elevated baseline ALP without ALP response after the first injection of radium‐223 had significantly worse OS when compared to all other patients (median OS 7.9 vs. 15.7 months, HR 2.56, 95% CI 1.73–2.80, *p* < 0.001).^25^ Thus, ALP response could be used to indicate the clinical benefit of radium‐223 treatment.

The median baseline PSA and ALP levels were lower in our study than in the ALSYMPCA trial (PSA 92 μg/L and ALP 122 U/L vs. PSA 146 μg/L and ALP 211 U/L). In our study, PSA decreased in 20% of patients (32 pts) during treatment when compared to baseline PSA value. In ALSYMPCA, 16% of patients had 30% or greater reduction in PSA at week 12 in the radium‐223 group. We found that very low baseline PSA levels (<20 μg/L) and the decrease in PSA level during radium‐223 treatment correlated with improved OS although the analyses showed that the magnitude of PSA progression had no statistically significant effect on the OS. Moreover, high PSA value (≥100 μg/L) before radium‐233 treatment and the short interval (<1 year) from the diagnosis of metastatic disease to the initiation of radium‐223 correlated with shorter overall survival, reflecting the more aggressive nature of the disease. The mechanisms causing low rates of PSA response with radium‐223 treatment are unclear. Adequate evaluation of the individual clinical benefit from radium‐223 is commonly challenging due to, for example, the PSA flare phenomenon after the initiation of radium‐223 therapy. In our study, we observed PSA flare followed by decreasing PSA in 8.1% of patients (13 pts). In the earlier study, the incidence of PSA flare has been reported to be 20.2%.^26^

In our study, we also focused on pain response during radium‐223 treatment. Over half of our patients (57%) achieved pain relief with radium‐223 and the pain relief during radium‐223 correlated with improved survival. Earlier prospective^27^ and retrospective^17^, ^20^ studies have reported similar pain response results. The quality of life was also reported to be better in the radium‐223 group compared to placebo in ALSYMPCA trial.^15^ Interestingly, a recent post hoc analysis of the phase III Proselica trial^28^ analyzed pain progression at the initiation of cabazitaxel treatment and found that the pain progression was associated with aggressive disease and shorter survival time compared to the radiological or PSA progression (12.0 vs. 16.8 vs. 18.4 months, respectively, *p* < 0.001). Pain response could be a potential indicator of the clinical benefit from radium‐223 and a marker of the aggressive course of mCRPC. Currently, there are ongoing prospective studies evaluating the effect of radium‐223 treatment to the pain response and quality of life (NCT04681144, NCT02398526).

There are several limitations in our study. Due to the retrospective nature of this study, there was no centralized analysis of radiological imaging or uniform assessment of disease progression, and no systematic questionnaires of the pain and quality of life during radium‐223 therapy were used. Other limitations were the incomplete baseline data of few individual patients due to a different health database at another hospital and a risk for missing and erroneous data entry. Our study cohort comprised also a quite homogenous Finnish Caucasian population, and we thus support further study across a wider variety of ethnicities. An adequate evaluation of the treatment response in prostate cancer with CT and bone scintigraphy bone metastases is challenging and currently there is no optimal imaging method to evaluate the clinical benefit from radium‐223 therapy. Automated Bone Scan Index (aBSI) has been suggested to be potential in evaluating the radiological response to radium‐223 in mCRPC patients, but more studies are needed.^29^ PET‐CT‐ or MRI‐based imaging methods are more sensitive than traditional CT and bone scintigraphy in detecting and evaluating bone metastases and could help to evaluate response.^30^, ^31^

Optimal patient selection of radium‐223 treatment remains challenging. In addition to clinical parameters, molecular features of tumors may dictate response to radium‐223 and may eventually help select patients that benefit of radium‐223. A retrospective study of 93 radium‐223 treated mCRPC patients found that mutations in DDR genes were predictive of better OS when compared to the DDR negative patients (median OS 36.3 vs. 17.0 months; HR 2.29; *p* = 0.01).^32^ Similar tendency was found in a study reported earlier showing OS of 36.9 months in patients with DDR mutations when compared to 19.0 months in patients without DDR mutations (HR = 3.3; *p* = 0.11)^33^ and prospective evaluation is ongoing in mCRPC (NCT04489719). DDR mutations are found in 20%–30% of patients with CRPC,^34^ but unfortunately the DDR mutation status of tumors in our cohort is not known. Overall, prospective evidence is anticipated regarding clinical or possible molecular predictive markers of radium‐223 response.

## 5 CONCLUSION

Our real‐world findings are in concert with the earlier randomized ALSYMPCA trial and few retrospective studies which show that radium‐223 is generally well tolerated although hematological toxicity due to the treatment or disease progression is commonly observed during therapy.^14^, ^17^, ^18^, ^21^, ^35^ In our cohort PSA decrease, ALP normalization and pain relief seem to correlate with improved survival. We advocate, however, that ALP and pain response should be studied in prospective settings as additional factors to define clinical benefit of radium‐223 while PSA and radiological response seem to be imperfect. Prospective studies are also needed for optimal patient selection and timing of radium‐223 treatment.

## AUTHOR CONTRIBUTIONS

Writing—original draft preparation: A.H. and M.S.; Writing—review and editing: A.H., T.U., K.M., P.A., H.M., and M.S.; Data collection: A.H. and O.K.; Statistical data analysis and visualization: A.H. and E.L.; Supervision: K.M., H.M., and M.S.; Project administration: M.S. All authors have participated in interpretation of data and read and agreed to the published version of the manuscript.

## FUNDING INFORMATION

This study has been funded in part by Finnish State Governmental Research Grant No. 13108 to Turku University Hospital and grants for A.H. from University of Turku and Department of Oncology, Turku University Hospital.

## CONFLICT OF INTEREST

AH, EL, PR, JS, and PA have nothing to disclose. OK had received conference participation, consultation, and lecture fees from Bayer, Ipsen, BMS, MSD Finland, Sanofi Genzyme, Lilly, Pierre Fabre, Roche, Merck, Jansen. TU had received conference participation, consultation and lecture fees from Amgen, Astellas, Bayer, BMS, Janssen, Orion, and Sanofi, and research funding from Bayer, Janssen, and Orion. MJ has been supported by Roche, Pierre Fabre, Amgen, MSD, and Abbvie for conference participation costs, and had received consulting or advisory honoraria from Amgen, Astra Zeneca, BMS, Ipsen, Merck, MSD, Novartis, Pfizer, Sanofi, and Sobi. HM has received research funding (not this study) from Finnish Cancer Foundations, Blue Earth Diagnostics, Merck, Philips, and Roche, and consultant fees from BMS, GSK, Jansen, MSD Finland, and Roche. KM had received consulting or advisory honoraria from Astellas, Bayer, Bristol‐Myers Squibb, Ipsen, Merck Sharp & Dohme, Merck–Pfizer alliance, Novartis, Roche, and Sanofi. MS has been supported by Pfizer, Novartis, BMS, Pierre Fabre, Roche, and Lilly for conference participation costs and received consultant fees from MSD, BMS, Roche, and Ipsen.

## Data Availability

N/A
